# Phase 1 dose-escalation study to evaluate the safety, tolerability, pharmacokinetics, and anti-tumor activity of FCN-159 in adults with neurofibromatosis type 1-related unresectable plexiform neurofibromas

**DOI:** 10.1186/s12916-023-02927-2

**Published:** 2023-07-03

**Authors:** Xiaojie Hu, Wenbin Li, Kang Zeng, Zhongyuan Xu, Changxing Li, Zhuang Kang, Shenglan Li, Xin Huang, Pu Han, Hongmei Lin, Ai-Min Hui, Yan Tan, Lei Diao, Ben Li, Xingli Wang, Zhuli Wu, Xiaoxi Lin

**Affiliations:** 1 Department of Plastic and Reconstructive Surgery, Shanghai Ninth People’s Hospital, Shanghai Jiao Tong University, School of Medicine, 639 Zhizaoju Road, Shanghai, 200011 China; 2 Department of Neuro-Oncology, Cancer Center, Beijing Tiantan Hospital, Capital Medical University, 119 South Fourth Ring West Road, Fengtai District, Beijing, 100070 China; 3 Department of Dermatology, NanFang Hospital Southern Medical University, 1838 North Guangzhou Avenue, Guangzhou, Guangdong 510515 China; 4 Clinical Pharmacy Center, Nanfang Hospital Southern Medical University, 1838 North Guangzhou Avenue, Guangzhou, Guangdong 510515 China; 5 Beijing Fosun Pharmaceutical Research and Development Co., Ltd, 1289 Yishan Road, Shanghai, 200233 China; 6 Fosun Pharma USA Inc., 91 Hartwell Ave Suite 305, Lexington, MA 02421 USA; 7 Present Address: EnCureGen Pharma, 9 Yayingshi Road, Guangzhou, 510525 China; 8 Shanghai Fosun Pharmaceutical Development Co., Ltd, 1289 Yishan Road, Shanghai, 20033 China

**Keywords:** Neurofibromatosis-1, FCN-159, MEK1/2 inhibition

## Abstract

**Background:**

Surgery is a common treatment strategy for patients with neurofibromatosis type 1 (NF1)-related plexiform neurofibroma (PN) and has limited efficacy. FCN-159 is a novel anti-tumorigenic drug via selective inhibition of MEK1/2. This study assesses the safety and efficacy of FCN-159 in patients with NF1-related PN.

**Methods:**

This is a multicenter, open-label, single-arm, phase I dose-escalation study. Patients with NF1-related PN that was non-resectable or unsuitable for surgery were enrolled; they received FCN-159 monotherapy daily in 28-day cycles.

**Results:**

Nineteen adults were enrolled in the study, 3 in 4 mg, 4 in 6 mg, 8 in 8 mg, and 4 in 12 mg. Among patients included in dose-limiting toxicity (DLT) analysis, DLTs (grade 3 folliculitis) were reported in 1 of 8 patients (16.7%) receiving 8 mg and 3 of 3 (100%) patients receiving 12 mg. The maximum tolerated dose was determined to be 8 mg. FCN-159-related treatment-emergent adverse events (TEAEs) were observed in 19 patients (100%); most of which were grade 1 or 2. Nine (47.4%) patients reported grade 3 study-drug–related TEAEs across all dose levels, including four experiencing paronychia and five experiencing folliculitis. Of the 16 patients analyzed, all (100%) had reduced tumor size and six (37.5%) achieved partial responses; the largest reduction in tumor size was 84.2%. The pharmacokinetic profile was approximately linear between 4 and 12 mg, and the half-life supported once daily dosing.

**Conclusions:**

FCN-159 was well tolerated up to 8 mg daily with manageable adverse events and showed promising anti-tumorigenic activity in patients with NF1-related PN, warranting further investigation in this indication.

**Trial registration:**

ClinicalTrials.gov, NCT04954001. Registered 08 July 2021.

**Supplementary Information:**

The online version contains supplementary material available at 10.1186/s12916-023-02927-2.

## Background

Neurofibromatosis type 1 (NF1) is an autosomal dominant neurocutaneous disorder characterized by multiple café au lait spots, neurofibromas, axillary freckling, Lisch nodules, osseous lesions (such as sphenoid wing dysplasia or long-bone dysplasia), and optic pathway glioma [1]. The average life expectancy of an individual with NF1 is reduced by approximately 15 years compared to the general population [2, 3]. Globally, the incidence of NF1 is approximately 1/2,500–1/3,000 [4]. Around 50% of those with NF1 have a family history of the disease; the other 50% have a de novo mutation in the *NF1* gene [5].

Whilst the clinical presentation and severity of NF1 is diverse, approximately 50% of individuals with NF1 present with plexiform neurofibromas (PNs) [6, 7]. PNs are nerve sheath tumors that are mostly benign, but because of their extent and location, they can cause deformity, loss of function, and death [7]. Additionally, some PNs are at risk of malignant transformation [8].

NF1 is caused by a lack of functional neurofibromin. Neurofibromin negatively regulates RAS activity in healthy individuals, but in individuals with NF1, RAS activity is dysregulated [5, 9]. Proteins encoded by the RAS oncogenes (*HRAS*, *KRAS*, and *NRAS*) are critical components in cell signal transduction and have key roles in numerous cell processes, such as cell proliferation, differentiation, and apoptosis [10, 11]. RAS is an upstream component of the MAPK (RAS/RAF/MEK/ERK) signal transduction pathway, which is heavily involved in regulating key cellular processes [12]. Activation of RAS small GTPase GTP/GDP exchange factors triggers the RAF/MEK/ERK kinase cascade via activation of MEK1/2, which in turn activates (phosphorylated and dimerized) ERK1/2. This induces target protein phosphorylation and regulation of other protein kinases that regulate cell function [12, 13]. Homeostatic negative feedback regulation of the RAS/RAF/MEK/ERK pathway is key in preventing uncontrolled cellular proliferation [13, 14]. Selective MEK1/2 inhibition may inhibit tumor growth in patients with tumors caused by RAS dysregulation; MEK1/2 are therefore potential actionable therapeutic targets for tumors occurring in patients with NF1 [12, 15].

The current treatment strategy for common manifestations of NF1 in adults, such as PN, is surgical resection. As PNs are highly infiltrative, surgical intervention can be challenging and of limited efficacy [16].

Mirdametinib and selumetinib are two orally administered small-molecule MEK1/2 inhibitors that are in clinical development for the treatment of NF1. Mirdametinib given to adult patients with NF1 and progressive PN at a dose of 2 mg/m^2^ twice daily resulted in a 42% (*n* = 8/19) response rate, with evidence of pain reduction [17]. The activity of selumetinib against not only NF-1-related PN, but also several cancers (including thyroid cancer [18], lung cancer [19] and melanoma [20]) has been observed, and selumetinib has been studied in children and adults with NF1-related PN. A phase I study showed that partial responses (PRs) were achieved in 71% (*n* = 17/24) of children treated with selumetinib at a dose of 20–30 mg/m^2^ twice daily [21]. A phase II trial showed that selumetinib resulted in PRs in 68% (*n* = 34/50) of patients [22], and since then selumetinib has become the first drug to be approved for NF1-related PN. As selumetinib is only approved in pediatric patients at the moment, there is a clear unmet clinical need for adults with NF1. Interim results from a phase II study of selumetinib in adults (NCT02407405) showed that 69% (*n* = 16/23) of patients achieved PRs [23].

FCN-159 is an orally available and highly potent selective MEK1/2 inhibitor, and a candidate targeted therapy for NF1-related PN tumors. It has a similar mechanism of action to mirdametinib and selumetinib [24]. In vitro study showed that FCN-159 is metabolized primarily by CYP3A4 and eliminated in a first-order elimination [25]. Preclinical studies using human colon cancer cells confirmed FCN-159 blocks the downstream MAPK pathway, preventing phosphorylation of MEK kinase and intracellular ERK. Anti-tumor activity was observed with FCN-159, inhibiting cell proliferation in selected RAS-mutant tumor cell lines, and tumor growth in nude mouse xenograft models (including human melanoma A375) [24].

A first-in-human study of FCN-159 has recently been conducted in patients with *NRAS*^mut+^ melanoma and was found to be well tolerated; no drug-related treatment-emergent adverse events (TEAEs) leading to discontinuation were observed, and promising, durable anti-tumor activity was reported [26]. Here we report data from a phase I/II clinical trial conducted to observe the safety and tolerability of FCN-159 and to determine the recommended phase II dose (RP2D) of continuous oral administration of FCN-159 in patients with unresectable NF-1-related PN. Preliminary anti-tumor activity was also assessed.

## Methods

### Study design

This is a multicenter, open-label, single-arm, non-randomized, phase I dose-escalation trial to evaluate the safety, tolerability, pharmacokinetic (PK), and anti-tumor activity of orally administered FCN-159 in adult patients with NF1 (NCT04954001). Phase I commenced with dose escalation in adult patients following a standard 3 + 3 design (Supplementary Table S1) to determine the maximum tolerated dose (MTD) of FCN-159 administered daily under fasting conditions (defined as no food or drink 1 h before and 2 h after each dose) in 28-day cycles. Study drug was dispensed at the study site and patient adherence was monitored by the records of drug dispensation and return. Patients were on treatment until PD, death, patient's voluntary withdrawal from study treatment, or study termination. The study was followed for 2 years after the last patient in. The starting dose was 4 mg, escalating in cohorts of at least three evaluable patients at 6 mg, 8 mg, and 12 mg until the MTD was determined. The starting dose in this study was determined based on available data from a phase 1a, first-in-human study of FCN-159 in patients with advanced *NRAS* ^mut+^ melanoma at the time of study design [26]. In that study, the dose of FCN-159 was escalated from 0.2 mg to 15 mg one daily, and no DLTs or SAEs were observed at 6 mg or lower as of October 20, 2020; systemic exposure to FCN-159 increased as the dose escalated [26]. Therefore, 4 mg was determined to be a safe starting dose for adults with NF1 in the present study. Dose expansion at a likely RP2D level enrolled approximately six patients. The highest dose level with a dose-limiting toxicity (DLT) incidence rate ≤ 33% (0/3 or 1/6 patients) was to be considered the MTD. If patients experienced grade ≥ 3 and/or unacceptable toxic events that were considered as at least possibly related to the study treatment, the study treatment was discontinued and supportive treatment was given in accordance with local treatment routines. If the toxicity returned to grade ≤ 2 within 28 days after the occurrence, and there was no progression on patients’ conditions or the investigator believed that patients could benefit from the study treatment, the original dose could be maintained or reduced after discussion with medical monitor. After completion of the dose escalation part of the study, PK, efficacy, safety, and tolerability data were analyzed to select the RP2D.

### Patients

Adult patients were eligible to enrol if they were aged between 18 and 70 years with confirmed NF1-related PN with a requirement of systematic therapy per Investigator’s judgment. The diagnosis of NF1 was made if at least one of the two diagnosis criteria was met: (1) positivity for NF1 germline mutation per Clinical Laboratory Improvement Amendments-certified laboratory (or equivalent) testing; (2) the presence of at least 2 of 7 manifestations or features according to the National Institutes of Health consensus criteria [1]. Patients must have been judged by the Investigator to have either PN unsuitable for surgery or have previously received surgical treatment with incomplete PN resection or relapse, and a measurable PN lesion of at least 3 cm in at least one dimension that was amenable to MRI analysis. Full inclusion and exclusion criteria are shown in Supplementary Table S2.

### Ethics, consent and permissions

The study was conducted in accordance with the principles of the Declaration of Helsinki and Good Clinical Practice guidelines and with the approval of the local Institutional Review Board/Independent Ethics Committee at the leading study site (Shanghai Ninth People's Hospital, approval number SH9H-2020-C25-6). All patients provided written informed consent to participate.

### Study endpoints

Primary endpoints of the study were the DLT incidence rate, MTD, and RP2D. Secondary endpoints included other safety assessments, imaging tumor response (complete response [CR], PR, stable disease [SD], or progressive disease [PD]), and PK parameters of FCN-159. Adverse events were graded per CTCAE v5.0.

### Safety and efficacy assessments

All patients underwent a complete medical history, physical examination, Eastern Cooperative Oncology Group (ECOG) assessment, and laboratory analysis of test results. Drug-related TEAEs, serious adverse events (SAEs), TEAEs of grade ≥ 3 toxicity, and TEAEs leading to drug discontinuation were reviewed on an ongoing basis. Efficacy was assessed by radiologic scans and clinical assessment. Tumor assessments per Response Evaluation in Neurofibromatosis Schwannomatosis criteria [27] were performed by investigators using MRI volumetric analysis every 4 cycles within the first 2 years (± 7 days) and every 6 cycles after 2 years (± 14 days) until PD, death, patient's voluntary withdrawal from study treatment, or study termination. MRI sequences of tau inversion recovery (STIR), T1- and T2-weighted sequences were chosen and the software of Extended Brilliance Workspace, SIGNA Premier and uExceed were used for MRI volumetric analysis. The same software was used for all tumor assessments at baseline and subsequent assessed timepoints of the same patient. Pain was assessed by an 11-point numerical rating scale (NRS-11).

### Pharmacokinetic assessments

In order to study the PK characteristics of FCN-159 in plasma, blood samples were collected pre-dose and 0.5, 1, 2, 3, 4, 6, and 10 h post dose on days 1 and 28 of cycle 1, and pre-dose only on days 2, 8, and 15 of cycle 1 and day 1 of cycle 2. Plasma PK parameters of FCN-159 in each dose group were analyzed with standard non-compartmental PK methods using Phoenix WinNonlin, v8.2. PK parameters included area under the curve (AUC)_(0-last)_, AUC_(0-∞)_, maximum plasma concentration (C_max_), time to maximum plasma concentration (T_max_), half-life (T_1/2_), oral clearance (CL/F), and volume of distribution (Vd/F) for the single-dose regimen and AUC_(0-tau)_, C_max_, T_max_, minimum steady-state plasma concentration (C_ss,min_), mean steady-state plasma concentration (C_ss, avg_), accumulation ratio for AUC (ARAUC), and apparent oral clearance at steady state (CLss/F) for the multiple dose regimen.

### Statistical analysis

The sample size for the dose-escalation study was estimated to be 24 based on the 3 + 3 method. Safety was analyzed in patients who received at least one dose of FCN-159 and had at least one safety assessment. DLT was assessed in the DLT analysis set, defined as patients who were enrolled in the dose-escalation study and met the DLT evaluation criteria. FCN-159 plasma concentration was analyzed in patients who received at least one dose of FCN-159, had at least one PK blood sample collection as scheduled, and had plasma concentration data for FCN-159. PK parameter analysis set comprised patients who received FCN-159 per protocol and had at least one PK parameter during the trial. Data were summarized using descriptive statistics.

## Results

### Patient disposition and baseline characteristics

**Fig. 1:**
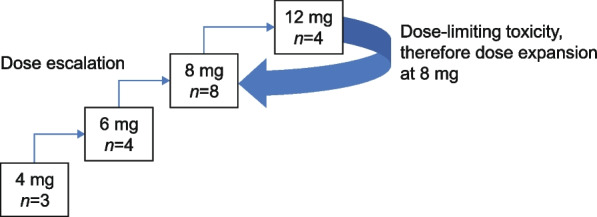
Study flowchart

Between 26th March 2021 and 4th November 2021, 23 patients were screened for the dose escalation study, and 19 were enrolled across four FCN‑159 dose groups: 4 (*n* = 3), 6 (*n* = 4), 8 (*n* = 8), and 12 mg (*n* = 4) (Fig. 1). The reasons for screening failure included withdrawal of informed consent (*n* = 2/4, 50%) and failure to meet study eligibility criteria (*n* = 1/4, 25%), and one patient withdrew in order to participate in phase II of this study (*n* = 1/4, 25%). The date of data cutoff was 1st December 2021.

The median age of the entire cohort (*n* = 19) was 26.0 years (range, 20–57 years; Table 1). Eleven (57.9%) were male and eight (42.1%) female. All patients had NF1 with associated PNs, and all had an ECOG performance status of 0. The most common type of neurofibroma-related complications at baseline were disfigurement (*n* = 10, 52.6%) and pain (*n* = 4, 21.1%). Eight (42.1%) patients had a positive NF1 germline mutation, one (5.3%) was confirmed negative. Of the eight (42.1%) patients with a known *NF1* zygotic type, all were heterozygotes. The most common locations for patients to present with PN were the face (*n* = 3, 15.8%), thoracic area (*n* = 4, 21.1%), and upper leg (*n* = 3, 15.8%). All PN localizations are shown in Table 1. The mean volume of target lesions at baseline was 47.8 cm^3^ (range, 2–4,670 cm^3^), and 15 (78.9%) patients also presented with measurable non-target lesions.

**Table 1:** Patient disposition and baseline characteristics

	**4 mg (*n* = 3) *n* (%)**	**6 mg (*n* = 4) *n* (%)**	**8 mg (*n* = 8) *n* (%)**	**12 mg (*n* = 4) *n* (%)**	**Total (*N* = 19) *n* (%)**
Median age, years (range)	25.0 (23–47)	32.0 (23–52)	23.5 (20–57)	30.0 (23–49)	26.0 (20–57)
Sex, *n* (%)					
Male	3 (100)	4 (100)	3 (37.5)	1 (25.0)	11 (57.9)
Female	0	0	5 (62.5)	3 (75.0)	8 (42.1)
Race, *n* (%)					
Asian	3 (100)	4 (100)	8 (100)	4 (100)	19 (100)
Median BMI, kg/m^2^ (range)	20.6 (20.6–25.7)	21.8 (21.2–28.1)	23.7 (19.2–25.8)	19.7 (18.1–20.2)	21.4 (18.1–28.1)
Median time from first diagnosis to first dose of treatment, months (range)	212.8 (164.8–356.8)	118.5 (0.8–418.4)	187.5 (0.4–600.0)	81.8 (0.5–121.4)	121.4 (0.4–600.0)
Neurofibroma-related complications, *n* (%)					
Disfigurement	2 (66.7)	1 (25.0)	4 (50.0)	3 (75.0)	10 (52.6)
Motor dysfunction	0	0	1 (12.5)	1 (25.0)	2 (10.5)
Pain	0	1 (25.0)	1 (12.5)	2 (50.0)	4 (21.1)
Vision	0	0	0	1 (25.5)	1 (5.3)
*NF1* germline mutation, *n* (%)					
Positive	0	1 (25.0)	4 (50.0)	3 (75.0)	8 (42.1)
Negative	0	0	0	1 (25.0)	1 (5.3)
Zygotic type, *n* (%)					
Heterozygote	0	1 (25.0)	4 (50.0)	3 (75.0)	8 (42.1)
Complication assignment, *n* (%)					
Motor	0	0	0	1 (25.0)	1 (5.3)
Others	3 (100)	4 (100)	8 (100)	3 (75.0)	18 (94.7)
PN locations, *n* (%)					
Orbit	1 (33.3)	0	0	0	1 (5.3)
Face	1 (33.3)	0	1 (12.5)	1 (25.0)	3 (15.8)
Anterior neck/upper airway	0	1 (25.0)	0	0	1 (5.3)
Posterior neck (cervical paraspinal)	0	0	1 (12.5)	1 (25.0)	2 (10.5)
Thoracic/paraspinal/chest wall	0	1 (25.0)	3 (37.5)	0	4 (21.1)
Posterior abdomen/pelvis (lumbosacral plexus)	1 (33.3)	0	0	1 (25.0)	2 (10.5)
Forearm	0	1 (25.0)	0	0	1 (5.3)
Thigh/upper leg	1 (33.3)	0	1 (12.5)	1 (25.0)	3 (15.8)
Foot	0	1 (25.0)	0	0	1 (5.3)
Others	0	0	2 (25.0)	1 (25.0)	3 (15.8)
Median volume of target lesions at baseline, cm^3^ (range)	158.3 (31–4,670)	18.7 (2–102)	39.7 (15–190)	421.0 (48–810)	47.8 (2–4670)
Non-target lesions at baseline, *n* (%)					
Yes	2 (66.7)	4 (100)	5 (62.5)	4 (100)	15 (78.9)
No	1 (33.3)	0	3 (37.5)	0	4 (21.1)

*Abbreviation*: *BMI* Body mass index

### Determination of dose-limiting toxicity

No DLT events were observed in the 4 mg and 6 mg doses. No DLT events were seen in the first three patients allocated to the 8 mg dose group. All three (100%) patients in the 12 mg dose group presented with folliculitis, therefore more patients were allocated to the expanded 8 mg dose group. One patient from the expansion phase that received the 8 mg dose presented with a DLT, folliculitis. This led to that dose group being expanded. The final proportion of patients presenting with folliculitis was 12.5% *(n* = 1/8). The MTD was therefore calculated to be 8 mg.

### Safety

Study-drug-related TEAEs were observed in all patients (*n* = 19, 100%), the majority of which were grade 1 or 2 in severity. The most common drug-related TEAEs were folliculitis (*n* = 9/19, 47.4%), stomatitis (*n* = 7/19, 36.8%), paronychia (*n* = 6/19, 31.6%), and increased blood alkaline phosphatase (*n* = 6/19, 31.6%). Twelve patients (63.2%) experienced gastrointestinal disorders that were reported to be drug related, of which stomatitis (*n* = 7/19, 36.8%) and mouth ulceration (*n* = 4/19, 21.1%) were the most common. Two (10.5%) patients experienced eye disorders: blurred vision (*n* = 1/19, 5.3%) and reduced visual acuity (*n* = 1/19, 5.3%). All drug-related TEAEs are shown in Supplementary Table S3.

Nine (47.4%) patients reported grade 3 drug-related TEAEs, including four patients experiencing paronychia and five experiencing folliculitis, which were the main cause of dose reduction (42.1%) and drug interruption (21.2%). Only one grade 3 drug-related TEAE was observed in patients receiving doses below the MTD (paronychia; 6 mg dose). One patient experienced an SAE during treatment that led to drug withdrawal. The SAE, rhegmatogenous retinal detachment, which was present at baseline, worsened (blurred vision developed) during the study and required elective surgical treatment, but the retinal condition did not get worse after FCN-159 administration; the event was subsequently considered unrelated to the study drug. No patients died during the study.

### Efficacy

**Fig. 2:**
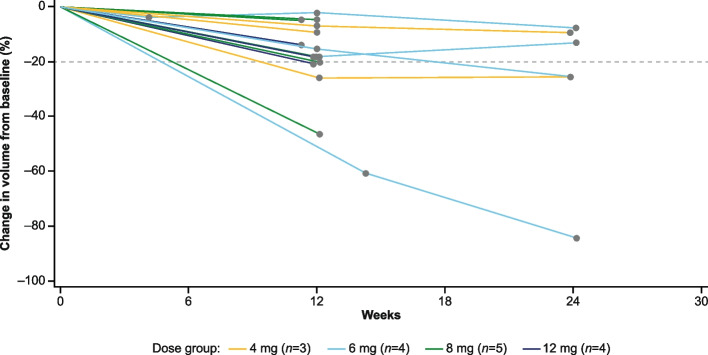
Spider plot showing changes in tumor burden over time

**Fig. 3:**
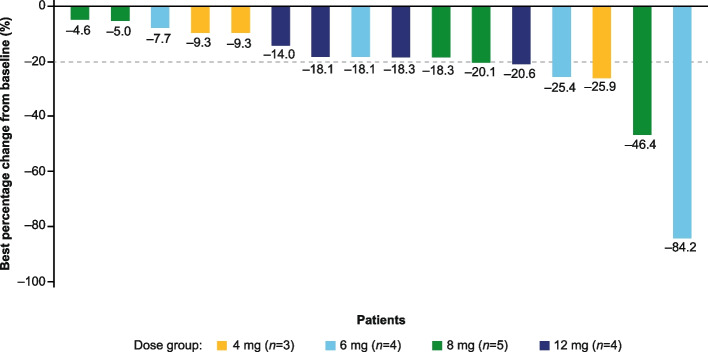
Waterfall plot showing the best percentage change from baseline of PN target lesions

The median relative dose intensity of drug exposure was 94.4% (range: 46.7–100.4). Of the 16 patients with at least one post-baseline tumor assessment, all (100%) had reduced tumor volume (Fig. 2). Six (37.5%) patients achieved PRs. All other patients had SD, and none experienced PD (Table 2). The largest reduction in tumor volume was 84.2% (Fig. 3). There was no correlation between tumor volume at baseline and the extent of tumor reduction. MRI images for two typical cases are shown in Supplementary Figure S1.

**Table 2:** Summary of best overall response based on Investigator assessments

	**4 mg (*n* = 3)**	**6 mg (*n* = 4)**	**8 mg (*n* = 5)**	**12 mg (*n* = 4)**	**Total (*n* = 16)**
Best overall response, *n* (%)					
Complete response	0	0	0	0	0
Partial response	1 (33.3)	2 (50.0)	2 (40.0)	1 (25.0)	6 (37.5)
Stable disease	2 (66.7)	2 (50.0)	3 (60.0)	3 (75.0)	10 (62.5)
Disease progression	0	0	0	0	0
Not evaluable	0	0	0	0	0

Patients with at least one post-baseline overall assessment of each respective dose group in the ITT population

*Abbreviation*: *ITT* Intent to treat

The results of pain score were limited at the data cutoff date. As it was important for patients with PN, we have provided results of pain as of 9 August 2022. Among patients with definite tumor pain (NRS-11 score ≥ 2 points) at baseline, 72.7% (16/22) of them had a reduction in pain intensity of at least 2 points at the assessment of cycles 4 and 5, which was considered clinically meaningful pain improvement; of them, 13 patients achieved complete pain relief at least once (the score reduced to 0). Pain scores decreased by an average of 2.4 points across all patients. At the dose group of 8 mg, 75% (15/20) of patients achieved meaningful improvement, with a mean pain score reduction of 2.5 points.

### Pharmacokinetics

**Fig. 4:**
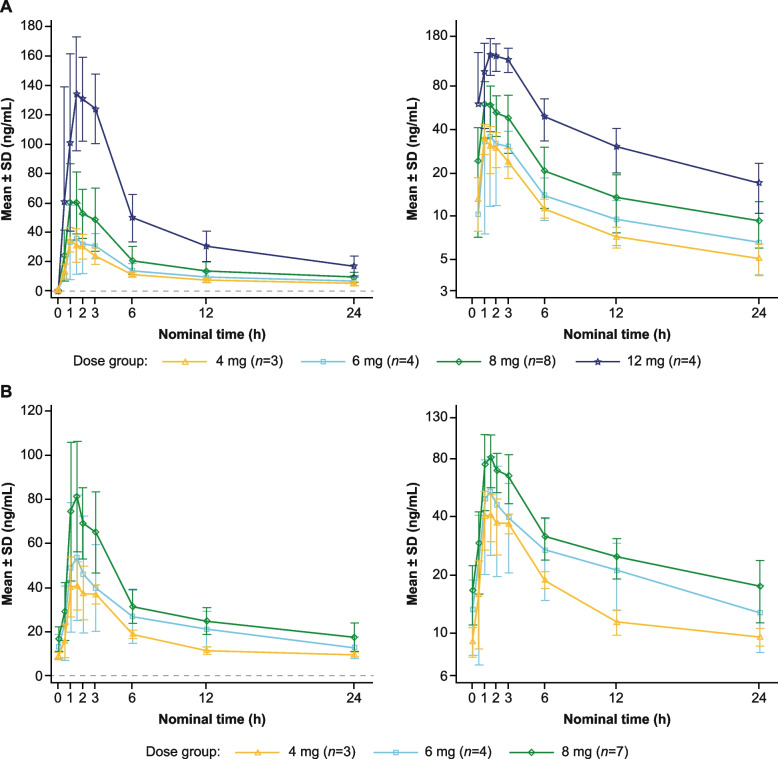
Plasma concentration of FCN-159 in **A**, single- and **B**, multiple-dose regimens. Graphs on the left of the panel are linear scale, and those on the right are semi-log scale

The mean plasma concentration–time profiles of FCN-159 in single- and multiple-dose levels from 4 to 12 mg are shown in Fig. 4. PK parameters of FCN-159 are shown in Supplementary Tables S4 and S5. As four participants in the 12 mg dose group had dose reduction during multiple dosing due to folliculitis, no PK profile is available for 12 mg at steady state. FCN-159 was absorbed rapidly, with a median T_max_ of 1.21–2.18 h across all dosing regimens and a mean terminal T_1/2_ from 11.4 to 16.2 h for single dose. The geometric mean C_max_ and AUC_tau_ of multiple doses over 4 mg to 8 mg ranged from 45.0 to 82.5 ng/mL and 392 to 723 h*ng/mL, respectively. C_max_ and AUC_tau_ at steady state were roughly dose proportional over the dose range 4–8 mg. The mean accumulation ratio of AUC from each cohort was 1.5–2.0 (Supplementary Table S6).

## Discussion

This phase I trial demonstrated that FCN-159 had low rates of DLT in adults with inoperable NF1-related PN and was associated with sustained reduction in PN tumor volumes. The MTD and RP2D were determined to be 8 mg daily with good tolerability and sustainable antitumor activity.

In the phase 1a study of FCN-159 in patients with advanced *NRAS* ^mut+^ melanoma, the RP2D was determined to be 12 mg based on a comprehensive assessment of safety, efficacy, and PK data [26]. In the present study among patients with NF1-related PN, the MTD was 8 mg because of the occurrence of DLTs at 12 mg, the RP2D was determined to be 8 mg after a comprehensive evaluation of safety data, antitumor activity, and PK characteristics of FCN-159. Unlike melanoma, which is a malignant tumor, NF1 is a benign tumor in nature, and therefore the difference in RP2D may be due to differences in the two patient populations, resulting in different tolerance. What’s more, a lower dose than that for patients with melanoma may improve treatment adherence and reduce the likelihood of dose interruption or discontinuation in patients with NF1-related PN, who are expected to survive longer than those with melanoma and need to take long-term medications to prevent tumor growth and relieve symptoms.

On the basis of this phase I trial, FCN-159 appears to have a favorable safety profile among similar drugs in its class. The first-in-human study of selumetinib had up to 71.4% of patients reporting AEs of CTCAE grade 3 or above, of which fatigue was the most common [28].This study shows 47.4% subjects taking FCN-159 experienced drug‑related AEs of CTCAE grade ≥ 3 across all dose levels, including four subjects occurring with grade 3 AEs at 12 mg. The RP2D dose of FCN-159, 8 mg, is well tolerated with the majority of AEs being grades 1 or 2. Among the most common drug-related TEAEs associated with FCN-159 were skin disorders, with grade 3 TEAEs presenting as folliculitis and paronychia. No other toxicities of grade 3 or higher were observed, except for skin conditions. Skin disorders also occurred frequently in selumetinib (62.5% of patients had acneiform dermatitis, [28]) and mirdametinib (78.8% of patients had rash [29]). Additionally, ophthalmologic toxic effects have been of particular concern for MEK inhibitors; blurred vision and visual disturbances have been observed with both selumetinib (visual function AEs in 17.9% of patients [28]) and mirdametinib (visual disturbance in 13.6% and blurred vision in 10.6% of patients [29]). In the present study, a minority of patients (10.5%) experienced treatment-emergent eye disorders in the form of blurred vision and reduced visual acuity, but these effects were mild. One individual did experience rhegmatogenous retinal detachment, but this was determined to be a result of an underlying condition at baseline and the retinal condition did not get worse after FCN-159 administration; as such, this event was not considered to be related to FCN-159.

Of the 16 patients whose tumor responses were evaluable, 37.5% had PRs. A previous study of mirdametinib showed a response rate of 42% in adults with NF1-related PN [17]. The response rate of selumetinib in the treatment of pediatric patients with NF1-related PN was approximately 40–70% [21, 22, 30, 31], whilst there is a lack of data regarding the effectiveness of selumetinib in adult patients. All patients had a reduction in tumor burden and zero patients had PD in this study. Unlike previous observations with imatinib [32], there was no correlation between baseline tumor size and extent of tumor reduction.

The RP2D of FCN-159 was determined as 8 mg daily, while it was 25 mg/m^2^ twice daily for selumetinib and 2 mg/m^2^ twice daily for mirdametinib. Due to its once-daily dosing regimen, FCN-159 may facilitate improved adherence, whilst the other two drugs require twice-daily administration. Furthermore, FCN-159 has a distinct PK profile compared with other MEK inhibitors. The C_max_ of FCN-159 was achieved after 1.21–2.18 h, which is slightly slower that selumetinib (1.00–1.10 h) and similar to mirdametinib (1.00–2.00 h) (26). The terminal half-life of FCN-159 ranged from 11.74 to 16.32 h, compared with 6.0–6.9 h for selumetinib and 4.6–18.0 h (average: 7.8 h) for mirdametinib, which requires twice-daily dosing [21]. The first-in-human study of FCN-159 in patients with *NRAS*^mut+^ melanoma reported a mean T_1/2_ of 32–57 h [26], which is higher still. The limited sampling duration in this study is likely what has led to a low estimation of the T_1/2_ in patients with NF1. Despite the relatively long T_1/2_ of FCN-159, a relatively low accumulation ratio was achieved, particularly compared with the MEK inhibitor trametinib [33]; this may be due to the two-compartmental PK profile of FCN-159. Systemic exposure of FCN-159 was roughly dose-proportional, unlike selumetinib, for which plasma concentration increase was less than dose-proportional [21]. In this phase I study, FCN-159 was administered under fasting conditions. A food effect study in healthy volunteers was conducted after the RP2D was declared. The results showed that food did not affect the pharmacokinetic profile of FCN-159 to a clinically meaningful extent compared with administration in the fasted state [34]. Therefore, patients in the following studies were allowed to take study drug with or without food.

This study is limited by its small study size and the narrow ethnic backgrounds of the trial patients. Although an NF1 mutation test was not mandatory according to the protocol, all patients underwent one genetic test after enrollment; these results are presented in Table 1. Additionally, further work could be done to establish mechanistic reasons why some tumors or patients were more responsive to FCN-159 than others.

This study has taken a robust 3 + 3 approach to determining the RP2D for FCN-159. It included a cohort of adults with PN for whom there are currently no treatment options, suggesting that FCN-159 will be of great benefit for adults with NF-1-related PN, for whom there is currently no approved drug. As FCN-159 is a MEK inhibitor, it is hoped that these results may contribute to the understanding of treatment to numerous cancers, not only NF-1-related malignancies. The RP2D is being taken forward to an ongoing trial, and additionally, these data are being used to inform a phase I pediatric study.

## Conclusions

To conclude, FCN-159 was well tolerated in patients with NF-1-related PN and demonstrated promising anti-tumor activity. The proposed RP2D for FCN‑159 was identified as 8 mg. The present findings support further investigation of FCN-159 as a treatment for patients with NF1–related PN, a group for whom the only intervention, surgery, is of limited efficacy.

## Supplementary Information

**Additional file 1: Supplementary Table S1.** Dose-escalation principle for adults. **Supplementary Table S2.** Study eligibility criteria. **Supplementary Table S3.** Frequency of Drug-related TEAEs. **Supplementary Table S4.** Summary of PK parameters (single dose). **Supplementary Table S5.** Summary of PK parameters (multiple dose). **Supplementary Table S6.** Dose-proportionality analysis – multiple-dose period. **Supplementary Figure S1.** Magnetic resonance images of plexiform neurofibroma (colored area) in (A) Patient 1 and (B) Patient 2 before (left) and after treatment (right).

## Data Availability

The data that support the findings of this study are available upon reasonable request to the corresponding authors Zhuli Wu and Xiaoxi Lin.

## Abbreviations

ARAUC: Accumulation ratio for AUC
AUC: Area under the curve
CL/F: Oral clearance
CLss/F: Apparent oral clearance at steady state
C_max_: Maximum plasma concentration
CR: Complete response
C_ss, avg_: Mean steady-state plasma concentration
C_ss,min_: Minimum steady-state plasma concentration
DLT: Dose-limiting toxicity
ECOG: Eastern Cooperative Oncology Group
ERK: Extracellular-signal regulated kinase
MAPK: Mitogen-activated protein kinase
MRI: Magnetic resonance imaging
MTD: Maximum tolerated dose
NF1: Neurofibromatosis type 1
PD: Progressive disease
PK: Pharmacokinetic
PN: Plexiform neurofibromas
PR: Partial response
RP2D: Recommended phase II dose
SAE: Serious adverse event
SD: Stable disease
T_1/2_: Half-life
TEAE: Treatment-emergent adverse event
T_max_: Time to maximum plasma concentration
Vd/F: Volume of distribution
