# Supplementary material for: Phase 1 dose-escalation study to evaluate the safety, tolerability, pharmacokinetics, and anti-tumor activity of FCN-159 in adults with neurofibromatosis type 1-related unresectable plexiform neurofibromas
Source: BMC Med. 2023 Jul 3;21:230. doi: 10.1186/s12916-023-02927-2 (PMC10318822; doi:10.1186/s12916-023-02927-2)

# Supplementary Appendix

**Supplementary Table S1.** Dose-escalation principle for adults

| **Dose groups** | 1 | 2 | 3 | 4 |
| --- | --- | --- | --- | --- |
| Increase from previous dose, %^a^ | - | 33 | 33 | 50 |
| Dose level, mg^a^ | 4 | 6 | 8 | 12 |
| Number of patients (estimated) | 3–6 | 3–6 | 3–6 | 3–6 |

Note: ^a^Tentative.

**File description**: Table outlining the dose escalation principle for the study.

**Supplementary Table S2.** Study eligibility criteria

| **Inclusion criteria** |
| --- |
| - Cohort 1: Age 18–70 years inclusive. Cohort 2: Age 2–18 years inclusive and able to swallow a whole tablet |
| - Diagnosis with NF1-related PN and symptomatic, with requirement of systematic therapy per investigator’s judgement. NF1 diagnosis is based on either genetic testing confirmation or clinical and imaging confirmation (26). |
| - Participant must be judged by the Investigator to be either unsuitable for complete surgical resection or the participant previously received surgical treatment, but that surgery left >15% of the primary lesion or the patient has since relapsed |
| - Participants must have a measurable lesion that is at least 3 cm in length in at least one dimension, and amenable to MRI for efficacy assessment |
| - For participants >16 years of age: Karnofsky performance level of ≥70%. For participants ≤16 years of age: Lansky performance score ≥70% |
| - Coagulation function: INR and APTT ≤1.5 ULN |
| - Participants (or legal guardians if younger than 18 years of age) are able to understand and voluntarily sign a written informed consent form |
| - For female patients or sexual partners of childbearing potential: agree to refrain from heterosexual intercourse or use contraception methods with a failure rate of <1% per year during treatment period and at least 30 days after the last administration of study drug and agree to avoid sperm donation. These restrictions in place for at least 90 days after the last dose |
| - Willing to avoid excessive sun exposure and use adequate sunscreen - Inclusion criteria specifically for phase I: Adequate organ and bone marrow function (absolute neutrophil count ≥1.5 × 10^9^/L, hemoglobin ≥9 g/dL, platelets ≥100 × 10^9^/L, serum total bilirubin ≤1.5 × ULN for age, ≤3.0 × ULN in participants with Gilbert’s syndrome, AST ≤2.5 × ULN, ALT ≤2.5 × ULN; albumin ≥3 g/dL, serum creatinine as per Supplementary Table S2 or a creatinine clearance or radioisotope GFR ≥60 ml/min/1.73 m^2^) |
| **Exclusion criteria** |
| - Patients who have received radiotherapy, surgery, or immunotherapy, or have participated in other interventional clinical trials within 4 weeks before the start of administration |
| - Patients who have received chemotherapy for NF1 within 3 months of enrollment |
| - Patients who have received treatment with any drug, biologic therapy, strong CYP3A4, CYP2C8 and CYP2C9 inhibitors or inducers, or moderate CYP2C8 and CYP2C9 inducers within 14 days of administration of FCN-159 |
| - Prior treatment with any other MEK1/2 inhibitors (phase II-specific) |
| - Participants with malignant tumors associated with NF1 requiring chemotherapy, radiotherapy, or surgery - Participants with current or history of other malignant tumors (excluding cured non-melanoma skin basal cell carcinoma, breast carcinoma *in situ* or cervix cancer *in situ*, and other malignant tumors without disease evidence for the past 5 years) |
| - Participants who are unable to undergo MRI examination and/or for whom MRI examination is contraindicated (e.g., due to prostheses, orthotics or dental appliances or due to interference with volumetric analysis of target PN on MRI) - Uncontrolled hypertension (despite medical therapy) - Participants with dysphagia, active digestive diseases, malabsorption syndrome, or other conditions that might affect the absorption of the study drug |
| - Previous or current RVO, RPED, glaucoma and other significant abnormality in ophthalmic examination - Interstitial pneumonia, including existing clinically significant radiation pneumonitis - Cardiac dysfunction or concomitant diseases meeting any one of the following conditions: |
| - - Three 12-lead ECG measurements were performed at the study site during the screening period, and the mean value of QTc in three measurements >470 milliseconds by calculated with the QTc formula of the instrument   - NYHA classification: class ≥3 congestive heart failure   - Clinically significant arrhythmia, including but not limited to complete left bundle branch block, second degree atrioventricular block   - Known concurrent clinically significant coronary artery disease, cardiomyopathy, severe valvular disease   - Ultrasound cardiogram performed during the screening showing LVEF <50% - Participants with active bacterial, fungal or viral infections, including active hepatitis B (hepatitis B virus surface antigen positive and hepatitis B virus DNA >1,000 IU/ml or meeting the study site's diagnostic criteria for active hepatitis B infection), hepatitis C (hepatitis C virus RNA positive), or human immunodeficiency virus infection (HIV-positive) - Pregnant or lactating women |
| - Known hypersensitivity to any MEK 1/2 inhibitors or their excipients |
| - Clinically significant condition that, in the opinion of the investigator, would preclude study participation or compliance with safety requirements - Inability to attend in-person appointments per current clinical site COVID-19 guidelines and restrictions |
| Abbreviations: ALT, alanine aminotransferase; APTT, activated partial thromboplastin time; AST, aspartate aminotransferase; ECG, electrocardiogram; GFR, glomerular filtration rate; INR, international normalized ratio; LVEF, left ventricular ejection fraction; NCI, National Cancer Institute; NYHA, New York Heart Association; RPED, retinal pigment epithelial detachments; RVO, retinal vein occlusion; ULN, upper limit of normal. |

**File description**: Table outlining the inclusion and exclusion criteria for the study.

**Supplementary Table S3.** Frequency of Drug-related TEAEs.

| Preferred term | 4 mg  (*N* = 3)  n (%) | 6 mg^a^  (*N* = 4)  n (%) | 8 mg^b^  (*N* = 8)  n (%) | 12 mg^c^  (*N* = 4)  n (%) | Total  (*N* = 19)  n (%) |
| --- | --- | --- | --- | --- | --- |
| Subjects with any drug-related TEAE | 3 (100) | 4 (100) | 8 (100) | 4 (100) | 19 (100) |
| Folliculitis | 0 | 1 (25.0) | 4 (50.0) | 4 (100) | 9 (47.4) |
| Stomatitis | 2 (66.7) | 2 (50.0) | 3 (37.5) | 0 | 7 (36.8) |
| Blood alkaline phosphatase increased | 0 | 1 (25.0) | 3 (37.5) | 2 (50.0) | 6 (31.6) |
| Paronychia | 1 (33.3) | 2 (50.0) | 3 (37.5) | 0 | 6 (31.6) |
| Alopecia | 0 | 1 (25.0) | 1 (12.5) | 3 (75.0) | 5 (26.3) |
| Blood bilirubin increased | 1 (33.3) | 2 (50.0) | 2 (25.0) | 0 | 5 (26.3) |
| Drug eruption | 1 (33.3) | 2 (50.0) | 2 (25.0) | 0 | 5 (26.3) |
| Protein urine present | 1 (33.3) | 2 (50.0) | 2 (25.0) | 0 | 5 (26.3) |
| Specific gravity urine increased | 1 (33.3) | 2 (50.0) | 2 (25.0) | 0 | 5 (26.3) |
| Urobilinogen urine increased | 2 (66.7) | 2 (50.0) | 1 (12.5) | 0 | 5 (26.3) |
| Blood fibrinogen increased | 0 | 0 | 0 | 4 (100) | 4 (21.1) |
| Blood lactate dehydrogenase increased | 1 (33.3) | 1 (25.0) | 1 (12.5) | 1 (25.0) | 4 (21.1) |
| Blood urea increased | 1 (33.3) | 2 (50.0) | 1 (12.5) | 0 | 4 (21.1) |
| Immunoglobulins increased | 2 (66.7) | 1 (25.0) | 1 (12.5) | 0 | 4 (21.1) |
| Mitral valve incompetence | 0 | 1 (25.0) | 2 (25.0) | 1 (25.0) | 4 (21.1) |
| Mouth ulceration | 0 | 0 | 1 (12.5) | 3 (75.0) | 4 (21.1) |
| QRS axis abnormal | 1 (33.3) | 0 | 0 | 3 (75.0) | 4 (21.1) |
| Blood creatine phosphokinase increased | 1 (33.3) | 0 | 2 (25.0) | 0 | 3 (15.8) |
| Breath odor | 0 | 2 (50.0) | 1 (12.5) | 0 | 3 (15.8) |
| Constipation | 0 | 0 | 1 (12.5) | 2 (50.0) | 3 (15.8) |
| Rash | 1 (33.3) | 1 (25.0) | 1 (12.5) | 0 | 3 (15.8) |
| Total bile acids increased | 1 (33.3) | 2 (50.0) | 0 | 0 | 3 (15.8) |
| Tricuspid valve incompetence | 0 | 1 (25.0) | 2 (25.0) | 0 | 3 (15.8) |
| Urinary occult blood positive | 0 | 1 (25.0) | 2 (25.0) | 0 | 3 (15.8) |
| pH urine decreased | 0 | 1 (25.0) | 2 (25.0) | 0 | 3 (15.8) |
| Alanine aminotransferase increased | 0 | 1 (25.0) | 1 (12.5) | 0 | 2 (10.5) |
| Aspartate aminotransferase increased | 0 | 2 (50.0) | 0 | 0 | 2 (10.5) |
| Beta globulin increased | 1 (33.3) | 1 (25.0) | 0 | 0 | 2 (10.5) |
| Blood uric acid increased | 1 (33.3) | 1 (25.0) | 0 | 0 | 2 (10.5) |
| Dyslipidemia | 1 (33.3) | 0 | 1 (12.5) | 0 | 2 (10.5) |
| Electrocardiogram abnormal | 0 | 0 | 0 | 2 (50.0) | 2 (10.5) |
| Epistaxis | 1 (33.3) | 0 | 1 (12.5) | 0 | 2 (10.5) |
| Hypocalcemia | 0 | 1 (25.0) | 1 (12.5) | 0 | 2 (10.5) |
| Menstrual disorder | 0 | 0 | 0 | 2 (50.0) | 2 (10.5) |
| Urine ketone body present | 0 | 0 | 2 (25.0) | 0 | 2 (10.5) |
| pH urine increased | 1 (33.3) | 0 | 1 (12.5) | 0 | 2 (10.5) |
| Abdominal pain | 0 | 0 | 1 (12.5) | 0 | 1 (5.3) |
| Acne | 0 | 1 (25.0) | 0 | 0 | 1 (5.3) |
| Acne pustular | 0 | 0 | 1 (12.5) | 0 | 1 (5.3) |
| Albumin globulin ratio decreased | 0 | 0 | 1 (12.5) | 0 | 1 (5.3) |
| Amylase increased | 0 | 0 | 0 | 1 (25.0) | 1 (5.3) |
| Anemia | 0 | 0 | 1 (12.5) | 0 | 1 (5.3) |
| Aortic dilatation | 0 | 0 | 1 (12.5) | 0 | 1 (5.3) |
| Asthenia | 0 | 1 (25.0) | 0 | 0 | 1 (5.3) |
| Beta globulin decreased | 1 (33.3) | 0 | 0 | 0 | 1 (5.3) |
| Blood pressure increased | 0 | 0 | 0 | 1 (25.0) | 1 (5.3) |
| Capillary disorder | 1 (33.3) | 0 | 0 | 0 | 1 (5.3) |
| Decreased appetite | 0 | 0 | 1 (12.5) | 0 | 1 (5.3) |
| Dermatitis acneiform | 0 | 0 | 1 (12.5) | 0 | 1 (5.3) |
| Diarrhea | 0 | 0 | 1 (12.5) | 0 | 1 (5.3) |
| Ejection fraction decreased | 0 | 0 | 1 (12.5) | 0 | 1 (5.3) |
| Electrocardiogram QT prolonged | 0 | 0 | 1 (12.5) | 0 | 1 (5.3) |
| Functional gastrointestinal disorder | 0 | 0 | 1 (12.5) | 0 | 1 (5.3) |
| Gamma-glutamyltransferase increased | 0 | 1 (25.0) | 0 | 0 | 1 (5.3) |
| Gingival pain | 0 | 0 | 1 (12.5) | 0 | 1 (5.3) |
| Hyperlipidemia | 0 | 0 | 0 | 1 (25.0) | 1 (5.3) |
| Hypertension | 0 | 0 | 1 (12.5) | 0 | 1 (5.3) |
| Hypoalbuminemia | 0 | 0 | 1 (12.5) | 0 | 1 (5.3) |
| Hypophosphatemia | 0 | 0 | 1 (12.5) | 0 | 1 (5.3) |
| Hypoproteinemia | 0 | 0 | 1 (12.5) | 0 | 1 (5.3) |
| Impetigo | 1 (33.3) | 0 | 0 | 0 | 1 (5.3) |
| Left atrial enlargement | 0 | 0 | 1 (12.5) | 0 | 1 (5.3) |
| Lipoprotein abnormal | 1 (33.3) | 0 | 0 | 0 | 1 (5.3) |
| Malaise | 1 (33.3) | 0 | 0 | 0 | 1 (5.3) |
| Muscular weakness | 0 | 0 | 1 (12.5) | 0 | 1 (5.3) |
| Oedema peripheral | 0 | 1 (25.0) | 0 | 0 | 1 (5.3) |
| Pain in extremity | 1 (33.3) | 0 | 0 | 0 | 1 (5.3) |
| Prealbumin decreased | 0 | 0 | 1 (12.5) | 0 | 1 (5.3) |
| Pruritus | 0 | 0 | 1 (12.5) | 0 | 1 (5.3) |
| Sinus arrhythmia | 1 (33.3) | 0 | 0 | 0 | 1 (5.3) |
| Sinus bradycardia | 1 (33.3) | 0 | 0 | 0 | 1 (5.3) |
| Sinusitis | 0 | 0 | 0 | 1 (25.0) | 1 (5.3) |
| Skin exfoliation | 0 | 0 | 1 (12.5) | 0 | 1 (5.3) |
| Supraventricular extrasystoles | 1 (33.3) | 0 | 0 | 0 | 1 (5.3) |
| Urinary sediment present | 0 | 0 | 1 (12.5) | 0 | 1 (5.3) |
| Urinary tract infection | 0 | 1 (25.0) | 0 | 0 | 1 (5.3) |
| Urticaria | 0 | 0 | 0 | 1 (25.0) | 1 (5.3) |
| Vision blurred | 0 | 0 | 1 (12.5) | 0 | 1 (5.3) |
| Visual acuity reduced | 0 | 1 (25.0) | 0 | 0 | 1 (5.3) |

Note: ^a^One participant reduced dose to lower dose level. ^b^Three participants reduced dose to lower dose level. ^c^Four participants reduced dose to lower dose level.

Abbreviation: TEAE, treatment-emergent adverse event.

**File description**: Table showing the classification and frequency of TEAEs reported during the study.

Supplementary Table S4. Summary of PK parameters (single dose)

| Dose group |  | AUC_0-t_ (h*ng/mL) | AUC_0-∞_ (h*ng/mL) | C_max_  (ng/mL) | T_max_^a^  (h) | T_1/2_  (h) | CL/F  (L/h) | V_d_/F  (L) |
| --- | --- | --- | --- | --- | --- | --- | --- | --- |
| 4 mg | N | 3 | 3 | 3 | 3 | 3 | 3 | 3 |
|  | Geomean | 250 | 370 | 36.0 | 1.43 | 16.2 | 10.8 | 253 |
|  | Geo CV (%) | 10.8 | 18.8 | 23.5 | 0.97–2.85 | 13.6 | 18.7 | 6.40 |
| 6 mg | N | 4 | 4 | 4 | 4 | 4 | 4 | 4 |
|  | Geomean | 262 | 388 | 38.1 | 1.48 | 11.4 | 15.4 | 254 |
|  | Geo CV (%) | 39.7 | 38.1 | 47.3 | 0.97–2.87 | 76.5 | 38.4 | 89.7 |
| 8 mg | N | 8 | 8 | 8 | 8 | 8 | 8 | 8 |
|  | Geomean | 453 | 661 | 64.2 | 1.21 | 15.4 | 12.1 | 269 |
|  | Geo CV (%) | 30.3 | 31.1 | 33.2 | 0.97–3.00 | 38.0 | 31.1 | 44.3 |
| 12 mg | N | 4 | 4 | 4 | 4 | 4 | 4 | 4 |
|  | Geomean | 1050 | 1330 | 147 | 2.18 | 11.7 | 9.03 | 153 |
|  | Geo CV (%) | 27.6 | 30.2 | 24.7 | 0.50–2.97 | 7.02 | 30.2 | 25.4 |

Notes: ^a^T_max_ is presented as median (min-max).

Abbreviation: Geomean, geometric mean; Geo CV, Coefficient of variation of geometric mean.

**File description**: Table outlining single dose pharmacokinetics.

Supplementary Table S5. Summary of PK parameters (multiple dose)

| Dose group |  | AUC_tau_  (h*ng/mL) | C_max,ss_  (ng/mL) | C_min,ss_  (ng/mL) | C_avg,ss_  (ng/mL) | T_max_^a^  (h) | CL_ss_/F  (L/h) | ARAUC_0-t_ | ARC_max_ |
| --- | --- | --- | --- | --- | --- | --- | --- | --- | --- |
| 4 mg | N | 3 | 3 | 3 | 3 | 3 | 3 | 3 | 3 |
|  | Geomean | 392 | 45.0 | 8.54 | 16.3 | 1.92 | 10.2 | 1.56 | 1.25 |
|  | Geo CV (%) | 12.0 | 16.1 | 13.1 | 12.0 | 0.97–2.95 | 12.1 | 1.92 | 21.2 |
|  |  |  |  |  |  |  |  |  |  |
| 6 mg | N | 4 | 4 | 4 | 4 | 4 | 4 | 4 | 4 |
|  | Geomean | 532 | 52.2 | 12.2 | 22.2 | 1.24 | 11.3 | 2.03 | 1.37 |
|  | Geo CV (%) | 53.7 | 69.7 | 48.7 | 53.8 | 0.97–1.93 | 53.7 | 23.3 | 39.9 |
|  |  |  |  |  |  |  |  |  |  |
| 8 mg | N | 7 | 7 | 7 | 7 | 7 | 7 | 7 | 7 |
|  | Geomean | 723 | 82.5 | 15.4 | 30.1 | 1.50 | 11.1 | 1.75 | 1.36 |
|  | Geo CV (%) | 23.3 | 25.3 | 32.4 | 23.1 | 1.00–2.87 | 23.2 | 18.6 | 20.0 |

Notes: ^a^T_max_ is presented as median (min-max).

ARAUC0-t = AUC0-t (steady state) / AUC0-t; ARCmax = Cmax (steady state) / Cmax; Geomean, geometric mean; Geo CV, coefficient of variation of geometric mean.

**File description**: Table outlining multiple dose pharmacokinetics.

Supplementary Table S6. Dose-proportionality analysis – multiple-dose period

| Dose range | PK parameter | Slope | 90% CI of slope |
| --- | --- | --- | --- |
| 4 mg ~ 8 mg | AUC_tau_ (h*ng/mL) | 0.90 | 0.36–1.43 |
|  | C_max,ss_ (ng/mL) | 0.92 | 0.25–1.59 |

Note:

Dose proportionality is assessed by fitting the relationship of the ln-transformed PK parameters (C_max_, AUC) to the ln-transformed dose using a power model log_e_(Y)=α+βlog_e_(Dose)+ε.

**File description**: Table outlining dose proportionality analysis for multiple dosing.

**Supplementary Figure S1.** Magnetic resonance images of plexiform neurofibroma (colored area) in **A**, Patient 1 and **B**, Patient 2 before (left) and after treatment (right)

**A**


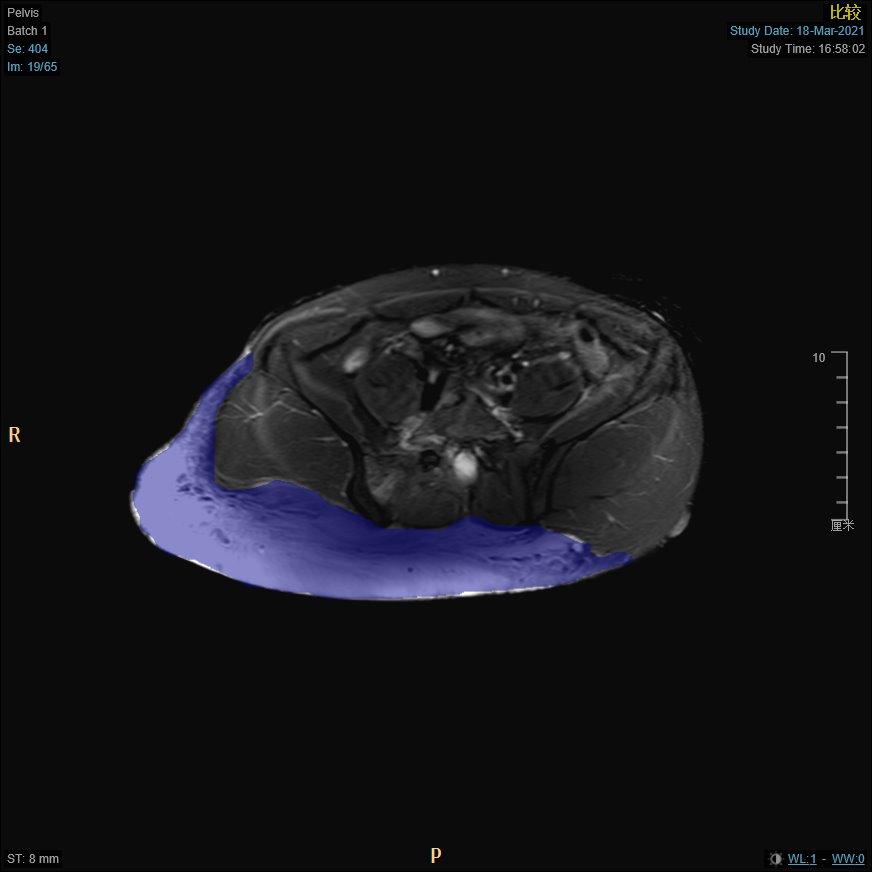

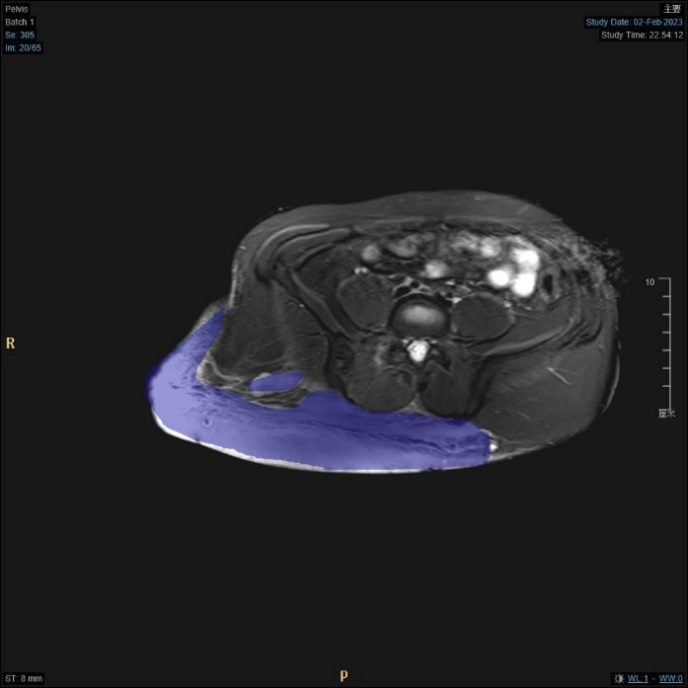


**B**


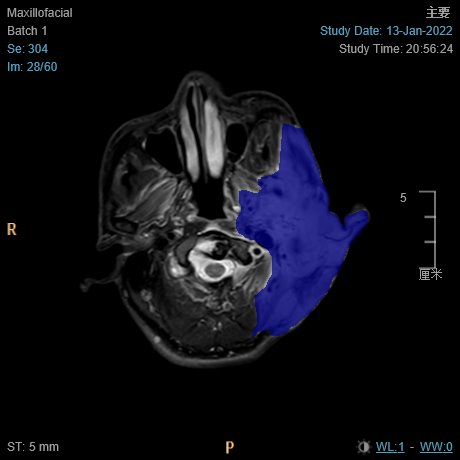

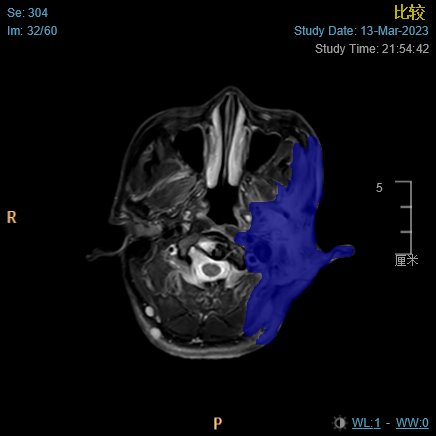

Supplement: Supplementary file 1 — Additional file 1: Supplementary Table S1. Dose-escalation principle for adults. Supplementary Table S2. Study eligibility criteria. Supplementary Table S3. Frequency of Drug-related TEAEs. Supplementary Table S4. Summary of PK parameters (single dose). Supplementary Table S5. Summary of PK parameters (multiple dose). Supplementary Table S6. Dose-proportionality analysis – multiple-dose period. Supplementary Figure S1. Magnetic resonance images of plexiform neurofibroma (colored area) in (A) Patient 1 and (B) Patient 2 before (left) and after treatment (right). [file 12916_2023_2927_MOESM1_ESM.docx]
